# Challenging the contest vs. scramble dichotomy in social competition: mixed conditions allow disparately ranked monkeys to get equivalent food but experiencing more competition still leads to risk-averse decisions

**DOI:** 10.3389/fnbeh.2025.1695267

**Published:** 2025-12-09

**Authors:** Erica J. Fowler, T. Jean M. Arseneau-Robar, Wilson Mutebi, Julie A. Teichroeb

**Affiliations:** 1 Department of Anthropology, University of Toronto Scarborough, Toronto, ON, Canada; 2 Wildlife Division, Nova Scotia Department of Natural Resources and Renewables, Kentville, NS, Canada; 3 Rwenzori Colobus Project, Nabugabo, Uganda

**Keywords:** contest competition, scramble competition, multi-destination foraging array, food quality, risk avoidance, foraging success

## Abstract

**Introduction:**

Food competition is a major cost to group living. Resources vary in quality, distribution, and handling times, exerting differing competitive regimes and varied effects on individual food intake depending on dominance rank.

**Methods:**

To investigate this interplay and the tipping points between purely contest and purely scramble scenarios, we conducted a field experiment on wild vervet monkeys (*Chlorocebus pygerythrus*), a species with linear, nepotistic intragroup dominance hierarchies. We baited a multi-destination foraging array with a mixture of clumped, preferred and less clumped, less preferred rewards to observe how individuals’ foraging decisions and route choices were affected by the presence and proximity of competitors. In contrast to previous experiments conducted with this group, rewards had minimal handling times and greater quantities to create a mix of scramble and contest competition.

**Results:**

We found that neither an individual’s dominance rank nor the frequency with which they faced competition from a dominant competitor significantly affected their overall foraging success, suggesting that we were successful in invoking scramble competition. All individuals, regardless of rank, generally chose to prioritize the best reward at the cost of a less efficient route and increased travel time. Nonetheless, encountering dominant competitors in a higher proportion of trials made focal individuals more likely to begin trials at the nearest, less preferred reward, rather than face contest competition for the preferred, more distant platform.

**Discussion:**

Our findings suggest that though greater scramble competition minimizes differences in food intake, risk avoidance still exerts powerful effects on the foraging route choices of those experiencing competition.

## Introduction

Foraging strategies vary based on a species’ diet, social group size, and the quality and distribution of available food patches (Garber et al., 2009; Janson and Goldsmith, 1995), which determine the amount and type of food competition experienced. Food competition is typically characterized as either scramble or contest (Nicholson, 1954). In contest competition (aka interference), competitors battle for control of resources, leading to dominance hierarchies, and allowing dominants to exclude subordinates from access (De la Fuente et al., 2019; Isbell, 1991; Sterck et al., 1997; Vogel, 2005). Contest competition is more likely to occur for high-quality, clumped resources that can be monopolized, often because they have a long handling time. High ranking individuals can also avoid some of the costs of finding (i.e., producing) or processing resources by challenging lower ranking group members and usurping food (i.e., scrounging and kleptoparasitism; De la Fuente et al., 2019; Vickery et al., 1991). In contrast, scramble competition (aka exploitation) occurs when resources cannot be, or are not worth, monopolizing (Isbell, 1991; Nicholson, 1954). This is usually because they are low-quality and/or distributed widely, and thus they allow lower ranking individuals to obtain an equal or greater share of resources, if they locate them first. Scramble competition affects all individuals in a group, and with equivocal resources it forces larger groups to search longer and over greater distances to fulfill every individual’s needs (Chapman and Chapman, 2000).

**Figure 1:**
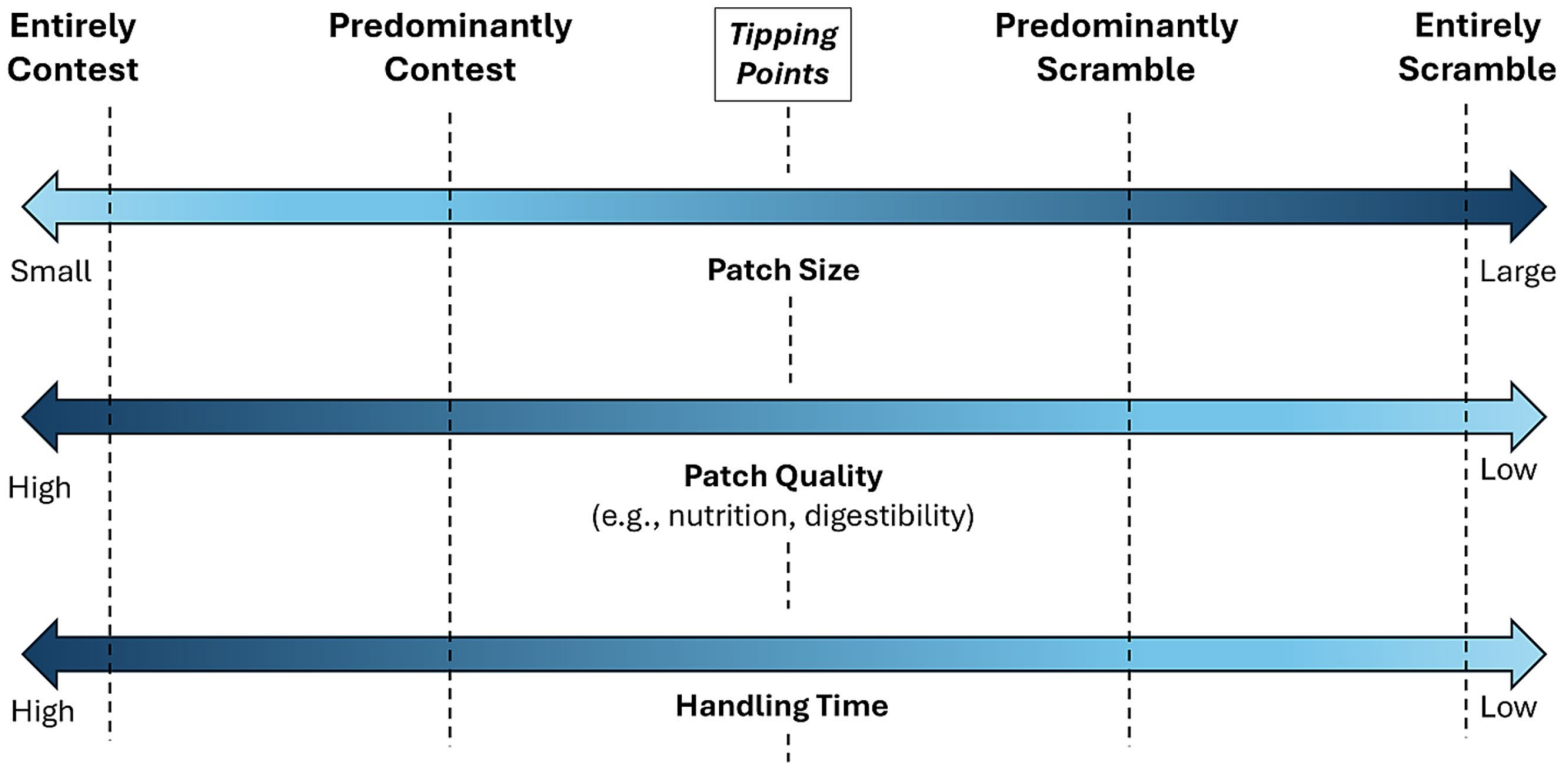
Depictions of patch-scale variables that, at their extremes, lead to contest and scramble competition for social individuals, but occur along a continuum. This leads to tipping points in the middle of the continuum where contest gives way to scramble and vice-versa.

Contest and scramble competition are usually described as distinct and treated separately (Janson and van Schaik, 1988). However, they can occur simultaneously, both within- and between-groups, and influence animal foraging strategies and social behavior in complex ways that are rarely investigated (Teichroeb and Sicotte, 2018). The existing literature has tended to focus on establishing the circumstances in which scramble or contest competition are predominant, with relatively little study of how the combination of contest and scramble competition can impact different individuals’ foraging strategies and rates of resource acquisition (but see De la Fuente et al., 2019). Indeed, scramble and contest competition as traditionally defined (Nicholson, 1954; Isbell, 1991), should be thought of as two ends of a continuum, where the variables leading to a food source eliciting one or the other (e.g., patch size, food quality, handling time) also vary along a sliding scale, so that there is a tipping point where predominantly scramble gives way to predominantly contest and vice versa (Figure 1).

In this study, we investigated the interplay and tipping point between scramble and contest competition with the aim of understanding how these different types of intragroup competition affect foragers’ decision making and foraging success. The number of variables that group-living animals need to consider before making foraging decisions are great and include both social and asocial considerations (Arseneau-Robar et al., 2022). For instance, competing group members can be closely monitored, so that foraging strategies can be modified based on competitor’s decisions [e.g., ravens (*Corvus corax*): Bugnyar and Heinrich, 2005; scrub jays (*Aphelocoma californica*): Clayton and Emery, 2001; Emery et al., 2004; eastern grey squirrels (*Sciurus carolinensis*): Teichroeb et al., 2024; chimpanzees (*Pan troglodytes*): Hare et al., 2001]. Foragers may also consider the social rank of competitors, and subordinate individuals may modify their foraging strategies when in the presence of dominants (e.g., Bicca-Marques and Garber, 2005; Ducoing and Thierry, 2003; Hare et al., 2001; Arseneau-Robar et al., 2022). If foragers do manage to arrive first at a resource (i.e., act as the producer), they will gain a ‘finder’s advantage’ where the amount they can profit (the ‘finder’s share’) (De la Fuente et al., 2019; Vickery et al., 1991) is dependent on the time elapsed between their discovery and the arrival of competitors. This time and the subsequent finder’s share is determined by their distance from the rest of their group, the handling time of the resource, and the individual’s dominance rank (De la Fuente et al., 2019; di Bitetti and Janson, 2001; Teichroeb et al., 2015; Vickery et al., 1991).

Besides these social considerations, Optimal Foraging Theory predicts that animals will evolve foraging strategies that minimize energy output and maximize energy intake (Charnov, 1976; Janson, 2007; Pyke, 2019) and empirical studies support this (e.g., Hazen et al., 2015; Hopkins, 2016). Some species, including many primates, have been shown to attempt to optimize the routes they take while foraging to reduce unnecessary travel time and distance while maximizing access to food resources (Cunningham and Janson, 2007; Janson, 2000, 2007; Kumpan et al., 2022). Optimal or near-optimal paths are often achieved by applying simple heuristics (i.e., rules-of-thumb). One common heuristic employed by primate foragers to avoid cognitive costs, the nearest neighbor rule (NNR), involves continually choosing the closest site that has not yet been visited (e.g., Janson, 2007; Teichroeb, 2015; Teichroeb and Smeltzer, 2018). Heuristic strategies can also vary by dominance rank. For example, when wild vervet monkeys (*Chlorocebus pygerythrus*) were presented with a foraging array with six identically baited sites, dominants used a cluster strategy to monopolize a set of nearby resources, while subordinates typically used a NNR to get what they could (Teichroeb, 2015). The presence of competitors can also change whether foragers choose to save energy by attempting to take the shortest path or bypass certain resources in favor of different or greater rewards farther away (Janson, 2007; Teichroeb and Aguado, 2016; Kumpan et al., 2019; Arseneau-Robar et al., 2022).

Here, we utilize a well-studied system, the foraging decision-making of vervet monkeys at Lake Nabugabo, Uganda, to delve more deeply into the interactions between contest and scramble competition and the outcomes in terms of foraging success and social behavior. We used an experimental multi-destination foraging array where we induced higher rates of scramble competition in comparison to earlier studies (e.g., Teichroeb, 2015; Teichroeb and Aguado, 2016; Teichroeb and Smeltzer, 2018; Arseneau-Robar et al., 2022) but maintained some elements that also allowed resource monopolization (i.e., contest competition). Our goal was to compare foraging outcomes and decision making with previous experiments in this system where contest competition predominated (e.g., Arseneau-Robar et al., 2022). Vervet monkeys are cercopithecines that are primarily frugivorous at Nabugabo (Cancelliere et al., 2018). They show linear dominance hierarchies, but also some tolerance towards group members depending on recent social interactions or previous social bonds (Borgeaud and Bshary, 2015). Male vervets disperse from the natal group and female dominance is based on matrilineal inheritance (Cheney and Seyfarth, 1983; Fairbanks and McGuire, 1986), although in some populations, dominance hierarchies have been found to be shallower and influenced by social dynamics other than matrilineal inheritance (Funk et al., 2024; Henzi et al., 2013). Studies show that the nature of foraging competition between conspecific vervets can change based on the environment, even in the same social group. For example, a study on free-ranging vervet monkeys in Kenya found that when food was clumped, higher ranking females had higher average food intake than lower ranking females, but these differences disappeared when food resources were scattered (Whitten, 1983). Similarly, later research on vervet and patas monkeys (*Erythrocebus patas*) found a greater number of agonistic interactions between females and a stronger linear dominance hierarchy in a habitat with clumped food resources, but less agonism and a weaker hierarchy in a habitat with more sparsely and randomly distributed resources (Pruetz and Isbell, 2000).

**Figure 2:**
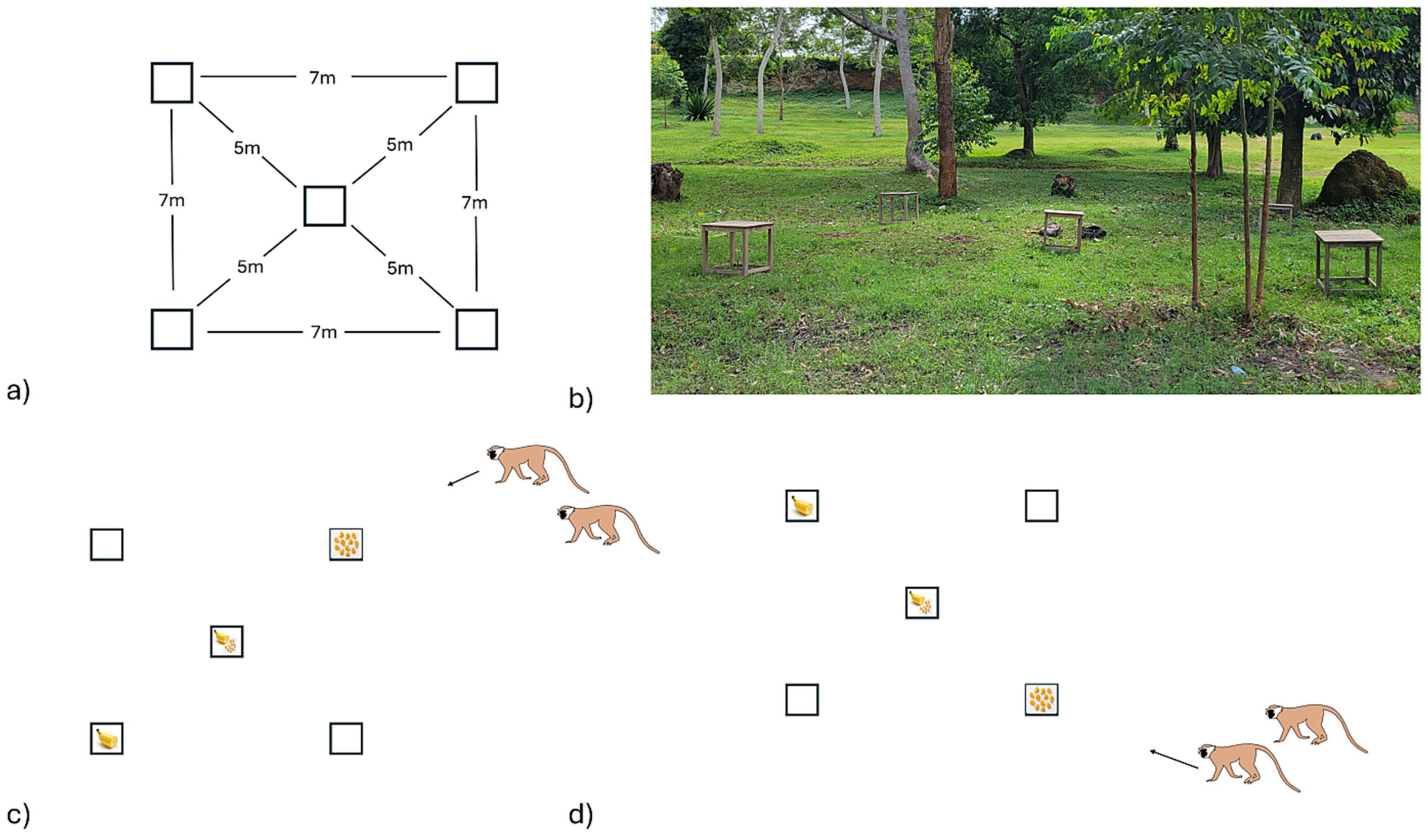
The experimental array used to assess the interplay between contest and scramble competition for food in vervet monkeys (*Chlorocebus pygerythrus*) at Lake Nabugabo, Uganda. Panel **(a)** shows the dimensions of the array, and **(b)** shows a photo of the array in its field location. Panels **(c,d)** show how the array was baited depending on the direction of approaching monkeys. The array was always baited in a straight line where the closest platform to approaching individuals contained 10–12 kernels of less-preferred corn (the lowest value platform), the middle platform was always baited with ¼ of an unpeeled banana and 10–12 kernels of corn (the highest value platform), and the platform furthest from approaching monkeys was baited with ¼ of an unpeeled banana (the middle value platform).

The foraging experiment conducted here allowed vervets to compete for access to platforms with different rewards, in a setup that presented an opportunity for scramble competition, and pitted their desire to minimize distance (e.g., Teichroeb, 2015; Teichroeb and Smeltzer, 2018), against their tendency to prioritize the best reward (e.g., Teichroeb and Aguado, 2016; Kumpan et al., 2019; Arseneau-Robar et al., 2022). We used an array of five platforms, where only three platforms were ever baited depending on the angle of the approaching monkey(s) (Figure 2). The three baited platforms were in a straight line, 5 m apart, such that the first platform approached contained several non-preferred food items, the middle platform had the largest and highest quality reward, and the final platform had a preferred but smaller reward. This experimental setup resembled that of Arseneau-Robar et al. (2022) in their study of the same vervet social group, but with key differences (i.e., decreased handling time and the inclusion of a second preferred reward) intended to increase rates of scramble competition. In this set up, prioritizing the best (favored) platform would necessitate bypassing the less preferred reward, and potentially losing it to an approaching competitor, but also necessitated traversing a greater distance to obtain all rewards if no competitor arrived. Although vervets have linear dominance hierarchies and high rates of contest competition (Pruetz and Isbell, 2000), the distance of 5 m between platforms and relatively low handling time of the food rewards presented an opportunity for scramble competition if dominant individuals were unable to block subordinate competitors from accessing the platforms.

We hypothesized that (H1) all vervets foraging without nearby dominant competitors would choose to begin at the nearest platform (lowest-quality reward) and access the three baited platforms in order of distance from the starting point to minimize travel distance (Teichroeb and Aguado, 2016; Arseneau-Robar et al., 2022). However, in trials with nearby dominant competitors, we expected dominants and subordinates to show different strategies. We hypothesized (H2) that the competition an individual experienced and dominance rank would both influence route choice. We predicted that (H2P1) higher ranking focal individuals would experience lower rates of competition at the experiment than subordinates and (H2P2) would be more likely to prioritize the preferred central platform as part of a ‘take-the-best’ strategy, while (H2P3) lower ranking individuals would experience greater competition and be more likely to start competitive trials at the less-contested nearest platform, due to the higher risk of aggressive confrontation at the central platform (Teichroeb, 2015). Regarding foraging success, we hypothesized that (H3) because our experiment was designed to induce more scramble competition, dominance rank would not be an important correlate of the amount of food obtained in a trial because subordinates would be able to consistently access the less-contested food rewards. Overall, we expected the vervets to establish their strategies within the first few trials in which they were participants and therefore (H4) did not anticipate previous experience in trials to significantly predict either (H4P1) choice of starting platform, or (H4P2) overall success obtaining food.

## Materials and methods

### Study ethics

The methods for this experiment were approved by the Uganda Wildlife Authority, the Uganda National Council for Science and Technology, and the University of Toronto Animal Care Committee. Animals were never handled or captured. Monkeys were able to leave the experiment site at any time.

### Study site and group

The study was conducted from May to July of 2022 on the Kasozi (KS) group, a previously habituated group of wild vervet monkeys (*Chlorocebus pygerythrus*) at Lake Nabugabo, Masaka District, Uganda. This period included the end of the rainy season one May before transitioning into the dry season for the rest of the study. The study group inhabits a fragmented forest in and around a rural farming village with numerous tourist camps. The group lives near human settlements and while they are not provisioned, they do forage on some crops and in garbage dump areas. At the time of study, KS group included 19–23 identified individuals, of whom 19 participated in trials as either focal individuals or competitors. The group included 2 adult males, 0–1 subadult males, 2 juvenile males, 8 adult females, 2 subadult females, and 5–8 infants. Seven individuals served as the focal individual in trials included in the analysis, including two adult males, four adult females, and one sub-adult female.

### Dominance hierarchy data collection

We conducted instantaneous scan sampling (Altmann, 1974) at 30-min intervals during full day follows and also recorded social interactions observed outside of scans *ad libitum*. We determined ordinal dominance rankings for individuals in KS group using daily observations of aggressive and submissive interactions between individuals, working from a baseline of hierarchical rankings known from previous studies of this group (Li et al., 2021; Arseneau-Robar et al., 2023; E. A. Smeltzer, Unpublished data). Displacements, avoidance, aggressive and submissive vocalizations, chasing, and physically aggressive behaviors (i.e., slaps, grabs, bites) were all used to determine dominance rank. The ability to usurp food resources during the experiment was further used to confirm dominance rankings. Dominance remained stable throughout the study. Although female vervet monkeys sometimes outrank males (Hemelrijk et al., 2020; Li et al., 2021; Young et al., 2017), adult males in this social group consistently outranked all adult and subadult females on an individual basis.

### Experimental setup

We used an array of five wooden platforms (0.75 m × 0.75 m surface, supported 0.75 m off the ground, Figure 2b) to conduct foraging testing trials. We arranged the platforms in a square configuration with the fifth platform in the center, measuring approximately 7 m × 7 m along the outer edges. The central platform was 5 m away from each outer platform (Figure 2a). The experimental site was in a small clearing behind a tourist camp in a lightly treed area with sparse undergrowth, providing good visibility of the platforms for both observers and participants in the vicinity. We placed the platforms within the study group’s home range and only members of KS group participated in the study; although members of a neighboring non-habituated group were sometimes present within 100 m of the experimental site, they were too fearful of humans to approach.

On each trial, we baited three of the five platforms in a diagonal formation (Figures 2c,d). Baiting only three platforms allowed for fast baiting, which meant that potential participants could remain close to the platforms during setup. This array also created a clear dichotomous choice of first platform for the monkeys, between prioritizing route efficiency or prioritizing preferred rewards. We baited the platforms between trials when all monkeys had moved at least 20 m away from the platforms. The first individual to reach the platforms in each trial was designated as the focal individual. Inter-trial intervals were typically brief and consisted of the few minutes needed to record data, reset the platforms, and bait them before the next trial. Trials continued until KS group left the experiment site, and KS group did not usually come to the experiment site more than once in a day. We baited platforms with a mixture of kernels of dried corn and pieces of unpeeled banana. The placement of the rewards was dependent on the position of the participants, with the least preferred reward always placed on the platform nearest to the monkey who was anticipated to be the focal (judged based on how close they were to the platforms and their dominance relative to other monkeys in the vicinity). The best reward was placed on the central platform, and the middling reward was placed on the furthest away platform diagonally opposite the anticipated focal monkey (Figures 2c,d). The monkeys consistently preferred bananas to corn (Arseneau-Robar et al., 2022). The central platform was always baited with a mixture of 10–12 pieces of corn and ¼ of a banana, constituting the best reward. The platform closest to the individual anticipated to be the focal was baited with a portion of corn (10–12 pieces), while the final platform diagonally opposite the focal, and furthest away, was baited with ¼ of a banana. We placed rewards directly on the platform rather than in containers to keep handling time low and provided more corn and a second preferred banana to encourage participation from more individuals and make it harder for dominants to control access to all preferred rewards, as in a previous study (Arseneau-Robar et al., 2022).

To keep track of the position and distance of the audience to participating monkeys, we mapped the study area, including all natural features, onto a 27 m × 27 m grid, with the position of the participants being marked on the grid between each change of position (by any individual) during a trial (Supplementary Figure S1). EJF and WM collected data on each monkey’s path through the platforms, the amount of food they consumed, as well as any social interactions during and between trials. We videotaped all trials to later confirm any uncertain variables. Ultimately, in our statistical models, the identity of audience members within 25 m was used as a measure of whether or not a dominant competitor was nearby during a trial because these individuals could approach the array quickly and interact with focal monkeys. However, we also recorded the identity of vervet audience members present within 100 m of the experiment site at the start of a trial to assess the potential competitors a focal individual may have observed.

We scored the total food obtained in a trial out of a total of six units of food available in each trial, overall encompassing two quarter pieces of a banana and two portions of corn (with the corn sometimes split into smaller fractions of a portion when it was shared between multiple individuals). Banana pieces were weighted as twice the value of a portion of corn, due to the clear preference of bananas over corn amongst the vervets and previous two-choice tests indicating that vervets would choose half a banana over a portion of corn unless the pieces of corn offered numbered around 40 or higher (TJMA-R, Unpublished data). As an example for this experiment, a monkey who consumed the entirety of the portion of corn placed on the nearest platform (1 food unit) and the portion of corn (1 unit) and banana (2 units) placed on the central platform would be scored as having consumed a total of four units of food out of the possible six.

We used the number of previous trials in which an individual had acted as the focal as a measure of their personal experience. Only individuals who participated as the focal in more than three trials were included in the analysis. Each focal’s first trial was also excluded to ensure that all individuals had an opportunity to become acquainted with the experimental setup before their choices were analyzed. Ultimately the study included seven focal individuals (*N* = 223 trials, *N* = 19 participants). To ensure that we were eliciting scramble and minimizing contest competition in this experiment, we made sure that resources had low handling times. We defined ‘handling time’ as the interval between the first touch of a food reward (or apparatus containing a food reward, as in Arseneau-Robar et al., 2022) to when the food items were all inserted into the mouth. Participants required minimal handling time to obtain an unpeeled quarter banana, averaging 1.7 s (*N* = 20) when in competition, because they were able to store the quarter banana in their cheek pouches to be eaten later and sometimes chose to consume it whole. Although the consumption of all individual kernels of corn in a unit had a somewhat slower average handling time of 6.0 s (*N* = 20), there was likewise no elaborate processing required. Compared to Arseneau-Robar et al.’s (2022) study, where the average handling time for that study’s banana reward located in a container was 6.7 s (*N* = 1,619), average banana handling time was significantly shorter in this study (*df* = 47, *t* = −17.1, *P* = > 0.001). The difference in the average handling time for a portion of corn (6.0 s) compared to the average handling time observed by Arseneau-Robar et al. (2022) (6.7 s) neared significance (*df* = 28. *t* = −1.69, *P* = 0.051).

In our final models, we analyzed competition at the array as an explanatory variable rather than separating competitive trials from non-competitive trials, as in some previous experiments (e.g., Teichroeb and Aguado, 2016; Kumpan et al., 2019). The experimental group was smaller during data collection for this experiment, and had fewer males, and individuals rarely traveled more than 75 m from the core of the group. This led to a dearth of solitary trials, and a complete lack of trials without a potential competitor within 100 m of the experimental site. We therefore did not separate solitary and competitive trials for comparison but instead assessed the impact of the proximity of competitors of higher rank. Data are available as Supplementary material.

### Data analyses

We created two statistical models, one to analyze the focal’s choice of first platform and one to assess the focal’s success obtaining food in a trial. To investigate route choice, we ran generalized linear models (GLM) using a logit link and we included in the final model (First Choice of Platforms Model): the focal’s number of previous trials (as a measure of experience), and the presence of a dominant competitor within 25 m of the platforms as predictive variables. We initially ran the model as a GLMM and also included the focal’s identity as a random effect, but the consistency in first choice of platforms across individuals, coupled with the relatively small number of individuals who participated as the focal, resulted in singular models that could not accurately estimate the limited variance in the individual identity variable. As such, we transitioned to a GLM that excluded the random effect for the First Choice of Platforms Model. Although the focal could theoretically have begun their trial at any of the three baited platforms, in practice, individuals always chose to travel first to either the central platform or the platform nearest to their starting position. Thus, the choice of starting platform was modeled as a binary choice [nearest (corn only) vs. central (banana + corn) platform] using a binomial distribution.

The focal’s success in a trial (Foraging Success Model) was modeled using linear mixed-effects models (LMM) with total food obtained (out of the possible six units of food, as detailed above) as the dependent variable. As the portions of corn were often split into smaller fractions due to being shared between multiple individuals, and the portions of banana were theoretically likewise divisible (although the splitting of a piece of banana was not observed in practice) total food obtained was modeled as a continuous variable. Predictor variables included presence of a dominant competitor within 25 m of the platforms at the start of the trial, previous experience as focal (number of previous trials as focal), choice of first platform, and individual identity (included as a random effect).

Initially, to assess what determined both choice of first platform and foraging success, we ran both models using the ordinal rank of the focal as a predictive variable. However, ordinal rank was found to be nonsignificant in all versions of both models. In the final models, we used a more direct measure of competition experienced by the focal individual: the proportion of total trials in which the focal individual encountered a dominant. This measure, previously employed by Arseneau-Robar et al. (2023), provides an approximation of competition experienced by an individual that can account for factors such as lower ranking individuals who deliberately avoided participating in trials with dominants or those that were tolerated by a dominant. While the focal’s rank and the proportion of total trials where that focal encountered a dominant competitor were highly correlated (*N* = 223, *r* = −0.753, *P* < 0.001), in some cases lower ranking individuals had a lower proportion of trials where they experienced dominant competition than did higher ranking individuals, and the two measures produced different results when incorporated into the statistical models.

The significance of predictor variables was set at a 0.05 alpha level and determined based on *z* scores (for the First Choice of Platforms Model) and likelihood ratio Chi-square tests comparing the original models to a succession of single term deletions. Confidence intervals were set at 97.5%. ANOVAs were used to compare both final models to null models that included only individual identity as a predictor. Models, comparisons, and diagnostics were run in R version 4.5.1 using the nlme, lme4, and DHARMa packages (Bates et al., 2015; Hartig, 2022; Pinheiro et al., 2023; R Core Team, 2025).

## Results

### Descriptive results

The focal monkey chose to start at the central platform (corn + banana) in 77.1% of trials or the platform nearest them (corn) in 22.9% of trials. No focal individuals ever chose to start at the outer platform farthest from their starting position. In 10.8% of trials, the focal individual chose to prioritize both bananas and bypassed all corn despite passing through the central platform where both corn and a banana piece were available. The alpha male (the highest ranking participant who experienced the least amount of competition across all trials) chose to access the central platform (corn + banana) first in a relatively high proportion of trials in which he was the focal (92.3%) and was the most likely to begin his trials at the central platform of all individuals included in the study. The focal individual who experienced the greatest amount of competition across this study chose to begin their trials at the central platform only 50.0% of the time—the lowest rate of trials beginning at the central platform of any study participant. Out of the possible six units of food a focal could obtain in each trial, the average units of food eaten by the focal was 4.010 (± SD 1.383). The highest ranking individual included in the study obtained an average of 4.685 (± SD 0.911) units of food in trials where they were the focal, while the lowest ranking individual obtained an average of 3.167 (± SD 1.693) units when acting as the focal.

### First choice of platforms model

An initial version of a GLM modeling the focal’s decision to begin either at the central platform (with the best reward) or at their nearest platform (Supplementary Table S1), found that higher ranking individuals were more likely to begin their trial at the central platform, although not significantly so (*N* = 223, X^2^ = 2.369, *P* = 0.124). In the final model (which substitutes rank for a more direct measure of the amount of competition experienced by the focal), the effects of the presence of a dominant competitor within 25 m, and previous experience as the focal, were found to be nonsignificant in the choice of initial platform using either *z* scores (First Choice of Platforms Model, see Table 1) or likelihood ratio tests (*N* = 223, X^2^ = 0.0261, *P* = 0.872, and *N* = 223, X^2^ = 1.205, *P* = 0.272, respectively). The only factor that significantly impacted the likelihood of a focal beginning their trial at the central platform was a measure of the amount of competition experienced across the whole experiment: the proportion of total trials they participated in where they encountered a dominant competitor (*N* = 223, X^2^ = 5.254, *P* = 0.022). Experiencing more competition (or a higher proportion of trials in which they encountered a dominant competitor) made focal individuals more likely to begin trials at the nearest platform, rather than the central platform.

**Table 1:** First choice of platforms model.

Responding variable	*B*	SE	*z*	*P*	Lower CI	Upper CI
Intercept	−2.178	0.662	–	–	–	–
Previous Experience	−0.006	0.006	−1.081	0.280	−0.018	0.005
Dominant >25 m	−0.064	0.396	−0.161	0.872	−0.867	0.694
**Proportion of trials w/ dominant**	**0.017**	**0.008**	**2.196**	**0.028**	**0.002**	**0.034**

Likelihood of the focal vervet monkey (*Chlorocebus pygerythrus*) traveling to the nearest, but less rewarding, platform first at Lake Nabugabo, Uganda. Significant effects are bolded.

An ANOVA comparing the GLMM version of the more complex First Choice of Platforms Model (with previous experience as focal in the current study, proportion of trials where the focal encountered a dominant competitor, presence of a dominant competitor within 25 m included as fixed effects, and identity of the focal individual included as a random effect) with a null model using only the focal’s identity (included as a random effect) as a predictor, found differences between the models that approached significance based on a likelihood ratio test (*P* = 0.059), and an increase in deviance of 7.428, suggesting that the First Choice of Platforms Model had a better fit than did the null model.

### Foraging success model

The focal individual’s total success in a particular trial, measured based on how much of the available six units of food were obtained, was modeled in LMMs using the maximum likelihood method (Table 2). The presence of a dominant competitor within 25 m of the platforms (*N* = 223, X^2^ = 36.666 *P* = <0.001), as well as choosing to travel to the nearest platform first (rather than to the central platform; *N* = 223, X^2^ = 6.577, *P* = 0.010) were both significant negative predictors of success. The proportion of trials where the focal encountered a dominant competitor did not predict the amount of food obtained (*N* = 223, X^2^ = 0.065, *P* = 0.799). An alternate version of the model substituting proportion of trials where the focal encountered a dominant with ordinal rank (Supplementary Table S2) found that higher ranking individuals did not obtain significantly more food (*N* = 223, X^2^ = 21.116, *P* = 0.291). When comparing the Foraging Success Model to a null model that included only individual identity as a random effect, an ANOVA found that the models were significantly different (likelihood ratio = 49.716, *P* < 0.0001).

**Table 2:** Foraging success model.

Responding variable	*B*	SE	*X^2^*	*P*	Lower CI	Upper CI
Intercept	3.786	0.543	–	–	–	–
**Dominant >25 m**	**1.326**	**0.206**	**−36.666**	**<0.001**	**0.903**	**1.714**
**Started at nearest platform**	**−0.494**	**0.194**	**6.577**	**0.010**	**−0.875**	**−0.118**
Proportion of trials w/ dominant	−0.002	0.007	0.065	0.799	−0.016	0.012
**Trial number**	**−0.003**	**0.001**	**−7.782**	**0.005**	**−0.005**	**−0.001**

Likelihood of focal vervet monkeys (*Chlorocebus pygerythrus*) obtaining the maximum available food in an experimental trial at Lake Nabugabo, Uganda. Significant effects are bolded.

An individual’s experience as the focal in previous trials (measured based on the number of trials previously completed wherein they acted as the focal) appeared to have a significant negative effect on feeding success (*N* = 223, X^2^ = 8.323, *P* = 0.004). However, previous experience was found to correlate highly with trial number (*r_s_* = 0.611). Replacing previous experience with trial number yielded the final version of the Foraging Success Model, wherein trial number was a significant negative predictor of success (*N* = 223, X^2^ = 7.782, *P* = 0.005).

## Discussion

Our investigation into the interplay between contest and scramble competition revealed nuanced strategies used by both dominant and subordinate vervets at Nabugabo where sensitivity to, and avoidance of, risk, played a strong role. Overall, our results did not support our first hypothesis (H1) that all vervets foraging without nearby dominant competitors would choose to begin at the nearest platform (lowest-quality reward) and access the platforms in order of distance to minimize travel distance (Teichroeb and Aguado, 2016; Arseneau-Robar et al., 2022). Across all trials, focal vervets were fairly consistent in their preference of beginning trials at the central and most rewarding platform (a take-the-best strategy), even though this choice required greater travel distance and bypassing the first lower-quality reward. By choosing the most rewarding platform first, individuals were granted immediate access to the banana and portion of corn on that platform. This choice also got them closer to the most distant, but second most rewarding, third platform containing the second banana, all of which made this route choice the most strategic and potentially most rewarding option despite the greater distance that needed to be traveled.

A perceived lack of reliability in the rewards offered by the two outer platforms could have contributed to the overall prioritization of the central platform. While the central platform always contained the most food and best reward, the peripheral platforms varied and could offer either a single preferred banana or the less desirable portion of corn. A portion of corn was always placed on the platform nearest to the monkey we assumed would become the focal, so which of the four outer platforms used depended on the positioning of the monkeys around the platforms; only the central platform was consistently baited with the same reward. Risk sensitivity theory suggests that a forager’s activity budget and energetic state can influence their willingness to accept risky foraging options, with individuals in a positive energy budget favoring less risky options (Caraco et al., 1980; De Petrillo and Rosati, 2021, but see Kacelnik and El Mouden, 2013). Research on foraging under risk and risk preference in primates has also demonstrated that social context, such as the presence of potential competitors or recent competitive interactions, can affect foragers’ propensities towards risk-seeking or risk-avoiding behaviors (De Petrillo and Rosati, 2021; Rosati and Hare, 2012; Zoratto et al., 2018). If the peripheral platforms were seen as inherently risky due to the variability in the rewards they offered, this uncertainty could create a stronger preference for the central platform if the vervets were employing risk-averse strategies. Our results suggest that vervet monkeys favor risk-averse strategies even at the cost of increased travel time and energy expenditure, and further systematic testing of this phenomenon could prove instructive.

In contrast to the predictions stemming from H1, the presence of a dominant competitor within 25 m did not have a significant impact on focal individuals’ route choices. However, in agreement with H2P2 and H2P3, individuals that experienced less competition overall in the experiment were more likely to prioritize the central platform most of the time, while individuals experiencing more frequent competition had a mixed strategy. They tended to have an even mixture of trials where they prioritized the central platform and where they began at the platform nearest to their starting position (the NNR). Our model showed that the more competition they experienced overall, the more they prioritized the nearest platform. Even though the peripheral platforms offered a lower probability of obtaining the preferred reward, this choice actually conformed with a risk avoidant strategy, as between two risks (i.e., a less rewarding food item and an altercation with a dominant) the vervets chose to avoid the more costly one (i.e., risk of injury during a fight or damage to a relationship as a result of a fight). Indeed, the choice of the closest platform allowed them to avoid agonistic confrontations at the favored central platform and reduce the amount of contest competition where higher ranking competitors would be more likely to prevail. In addition, it may also have allowed them to flee the study site more easily if there was risk of aggression because they were on the edge of the array.

Dominance rank and competition experienced in the experiment were highly correlated (supporting H2P1). However, somewhat unexpectedly, the tendency to alter first platform choice was not reflected when dominance rank was used as a predictive factor, but rather when the amount of competition experienced was used. This discrepancy may have occurred because some of the higher ranking females (particularly the highest ranking female) appeared more willing to engage in trials in which they faced dominant competition, even from the alpha male, while the lower ranking females and the subordinate male were usually in the vicinity but appeared more selective about their participation, possibly as part of an effort to avoid the risk of contest competition and aggressive encounters with dominants. Consequently, the highest ranking female experienced greater rates of competition than did some lower ranking individuals and, as in Arseneau-Robar et al. (2023), the focal’s ordinal rank was not always an accurate measure of how much competition an individual experienced.

The presence of a dominant competitor within 25 m of the array at the beginning of a trial did not significantly affect the likelihood of the focal individual choosing the central platform (rather it was the overall amount of competition experienced that was important). As such, it is possible that, after having decided to participate in a trial, monkeys were not influenced by the presence of audience members when choosing their first platform (perhaps assuming that the possibility of competition from a dominant always existed). Conversely, for dominant monkeys, they likely still perceived a risk from subordinates running in and snatching food quickly in spite of their lower rank (e.g., Joyce et al., 2023). As there were other members of the group visible within the vicinity in nearly all trials, and, in some cases, members of a neighboring vervet group as well, the focal vervets may have consistently anticipated competition across all trials. This could explain the consistency in route choice across all individuals; the potential for competition was always present, and it made little difference to the focal monkey whether it came from subordinates, dominants, or even members of another social group.

Foraging success was significantly impacted by our measures of experience (in opposition to H4P2), while route choice remained consistent over time (supporting H4P1). Our results showed that, somewhat surprisingly, focal individuals obtained less food in later trials regardless of an individual’s personal experience. We attribute this to the early inexperience of the dominant male in experimental regimes and his gradual improvement in performance as a competitor. The alpha male of KS group did not come from a habituated study group with experience in previous experiments, and initially, he took longer to approach the platforms than did other monkeys, gradually increasing his skill and participation. Indeed, when only trials where the alpha male served as a competitor were included, a type 2 *t* test found that focal individuals obtained significantly less food during the second half of the study in comparison to the first half (*n* = 107, *P* = 0.046).

We found mixed support for our third hypothesis (H3), which predicted no relationship between dominance rank and the amount of food obtained because we induced greater scramble competition. While the presence of a dominant competitor within 25 m of the platforms before a trial did hinder the focal’s ability to successfully obtain food and likely impacted their decision making (supporting H2P1 and H2P2), dominance rank and frequency of competition experienced by the focal did not significantly affect the amount of food obtained during a trial (supporting H3). This suggests that we successfully evoked scramble competition, and it played a prominent role in the actions of the vervets during this experiment. Indeed, the relative lack of foraging success of dominant individuals in this experiment contrasts with the results of a previous study on the same social group in which vervets competed for a series of rewards distributed across five platforms, each 5 m apart (Arseneau-Robar et al., 2022). That study utilized a greater number of platforms and also forced vervets to retrieve the favored banana from inside a container, increasing handling time and evoking more contest competition (Figure 1). In our experiment, the focal individual chose to prioritize the highest reward platform in 77.1% of trials (*n* = 223), in comparison to the 22% of trials (*n* = 1,028) observed by Arseneau-Robar et al. (2022). The much lower handling time to obtain food in the current study allowed individuals experiencing higher rates of competition greater opportunity to quickly obtain rewards before a dominant competitor could reach their platform. Indeed, when a more dominant individual was the focal, we observed some subordinate competitors quickly snatch a single banana before retreating, highlighting the frequent inability of dominant competitors to control access to all three feeding platforms. Unlike in Arseneau-Robar et al.’s (2022) study, there were very few trials where subordinate monkeys were not present in the immediate vicinity of the experimental site. This likely made it more difficult for dominants to control the area, particularly when there were multiple competitors attempting to gain access. The disparity in results between this study and Arseneau-Robar et al.’s (2022) aligns with previous research showing that resource size and handling time have a greater impact on the type and frequency of food competition than does the spatial distribution of resources; with mid-sized, high handling time resources that can be monopolized producing high rates of contest competition and aggression (Chancellor and Isbell, 2008; Hanya, 2009; Pruetz and Isbell, 2000).

While Arseneau-Robar et al.’s (2022) study was also conducted on KS group, the social dynamics and membership of this group had been greatly altered in the years between studies. At the time of the present study, KS contained only around half as many individuals as were present during the previous experiments. Even though KS group contained fewer individuals during our study, our trials had a significantly higher mean number of active competitors per trial (1.5; *n* = 223) in comparison to Arseneau-Robar et al.’s (2022) trials (mean of 0.6; *n* = 1,664; *t* test: *df* = 1885, *t* = 17.8, *P* = <0.001). The group included only two adult males, in contrast to the seven males who participated in Arseneau-Robar et al.’s (2022) study. The males who were present for the current study were also relatively new to KS group and, unlike some of the females, had not had the opportunity to participate in, or witness, any previous foraging experiments. This may have contributed to the comparatively lower amount of success and control experienced by the males in this study relative to that of Arseneau-Robar et al. (2022). Though females were lower ranking, their experience led them to be first to access the experiment, and some females were the main participants, which contrasts with earlier studies in this system where bolder males dominated platform access (Teichroeb, 2015; Teichroeb and Aguado, 2016). It should also be noted that the adult female with the lowest ordinal rank never participated in any trials, and that the next three lowest ranking females participated very rarely despite often being in the vicinity of the platforms while trials occurred. While mid- and higher-ranking females were able to employ scramble competition strategies to obtain similar success rates to more dominant females and males, the lowest ranking adults either had little opportunity to access the platforms or avoided the risk of direct confrontation with a dominant. Our findings echo similar results obtained in a foraging experiment comparing the finder’s share and foraging success of marmosets (*Callithrix jacchus*) of different rank when food resources were either clumped or scattered (De la Fuente et al., 2019). The authors found that when food resources were scattered, both high- and low-ranking individuals were able to act as producers, which increased overall feeding success, and that dominance rank only had a significant effect on feeding success in the case of the highest ranking individual. A study investigating the foraging dynamics of free-ranging dogs presented with a variety of feeding patches of varying sizes likewise observed a mixture of influences from both scramble and contest competition (Berghänel et al., 2025). Individuals’ ranks and social affiliations were found to be predictive of their feeding success, but aggression decreased as feeding group size increased over three individuals, suggesting that scramble competition played an increasingly large role as more competitors arrived.

Although this experiment had a much simpler setup with fewer reward sites, the results are in line with findings from Teichroeb (2015), where higher ranking vervets employed a cluster strategy (choosing the central platform first and getting closer to the second-best platform) while lower ranking vervets were more likely to use a NNR. In some cases, high handling time can make kleptoparasitism and contest competition more advantageous for higher ranking individuals, who can rely on subordinates to handle food processing before appropriating the extracted resources (Marshall et al., 2012; Vickery et al., 1991). In this study, the negligible handling time associated with the food rewards could be said to remove a potential advantage for high-ranking individuals. The platforms may also have been too far apart to allow dominant individuals to successfully monopolize them using a cluster strategy, despite attempting to. The tendency to prioritize the preferred central platform may be indicative of vervets employing a ‘take-the-best’ heuristic. This is defined as when a decision maker chooses between binary options based on the first piece of distinguishing information available, only seeking additional information if the first characteristic observed does not give an indication of which option is “best” (Gigerenzer and Goldstein, 1996). In this case, use of a “take-the-best” heuristic could influence vervets to target the best reward and ignore additional information about the relative advantages of the alternatives (i.e., which would have allowed a more efficient route through the platforms).

## Conclusion

Our experiment, which induced both contest and scramble competition, shows that both impact the decision-making of individual vervets and that tipping points between the two (Figure 1) lead to a mix of risk-aversive strategies. Dominant monkeys, who were unable to control resources entirely, as in a pure contest competition scenario, focused on acquiring the preferred, high-quality resources before subordinates could snatch them, showing a mix of behaviors typically seen in contest and scramble scenarios. Subordinates also chose the best resources when they could, but those that experienced more competition overall avoided costly confrontations with dominants by selecting the lower-quality, less preferred resource first more often. The least dominant animals were the most risk-averse and often strategically chose not to participate at all.

Additional research on the interplay between contest and scramble competition and the circumstances that produce variable behavioral strategies could provide further understanding of the decision-making of foragers and how social rank and relationships impact intragroup foraging competition. Future work should also examine what factors motivate participation from individuals in the vicinity of foraging experiments in the wild (as in Arseneau-Robar et al., 2023), which could prove insightful. The effects of an individual’s activity budget, social capital, or expectations of social tolerance were not examined in the present study but have been observed to affect foraging strategies (e.g., De Petrillo and Rosati, 2021; Garber et al., 2009; Li et al., 2021; Marshall et al., 2012; Trapanese et al., 2018) and could potentially impact success rates in similar foraging experiments, constituting ripe areas for future study.

The similarity in foraging success for individuals of disparate rank, that experienced different degrees of competition, indicates that our experimental setup created scramble competition and circumstances under which subordinates were able to gain benefits more like those of dominants compared to other studies in this system (e.g., Teichroeb, 2015; Arseneau-Robar et al., 2022). In this case, the short to negligible time elapsed between the focal individual’s discovery of a resource/reward (the finder’s advantage) and the arrival of competition appears to have been offset by minimal handling time and sufficient distance between platforms to allow for the successful use of scramble competition strategies. While social rank is undoubtedly a major influence on foraging decisions and success in the context of social groups, the results of this study support that food distribution and availability can overpower the influence of social rank and therefore merit careful consideration from researchers. Overall, our study demonstrates that, despite the importance of dominance rank in vervet monkey societies, their foraging strategies are flexible and context dependent.

## Data Availability

The raw data supporting the conclusions of this article are available as Supplementary materials. Further enquiries should be directed to the corresponding author(s).
