# Supplementary material for: Challenging the contest vs. scramble dichotomy in social competition: mixed conditions allow disparately ranked monkeys to get equivalent food but experiencing more competition still leads to risk-averse decisions
Source: Front Behav Neurosci. 2025 Dec 9;19:1695267. doi: 10.3389/fnbeh.2025.1695267 (PMC12722904; doi:10.3389/fnbeh.2025.1695267)
Supplement: Supplementary file 2 [file Supplementary_file_1.docx]

Supplementary Materials

**Challenging the contest vs. scramble dichotomy in social competition: mixed conditions allow disparately ranked monkeys to get equivalent food but experiencing more competition still leads to risk-averse decisions**

Erica J. Fowler^1^, T. Jean M. Arseneau-Robar^1,2^, Wilson Mutebi^3^ and Julie A. Teichroeb^1,*^

^1^Department of Anthropology, University of Toronto Scarborough, Toronto, ON, Canada

^2^Nova Scotia Department of Natural Resources and Renewables

Wildlife Division, Kentville, NS, Canada

^3^Rwenzori Colobus Project, Nabugabo, Uganda

^*^To whom correspondence should be addressed:

julie.teichroeb@utoronto.ca

**Table S1.** Likelihood of the focal vervet monkey (*Chlorocebus pygerythrus*) traveling to the nearest, but less rewarding, platform first at Lake Nabugabo, Uganda. Includes the focal’s rank as a predictor. Confidence intervals are set at 97.5%.

|  | ***B*** | **SE** | ***z*** | ***P*** | **Lower CI** | **Upper CI** |
| --- | --- | --- | --- | --- | --- | --- |
| Intercept | 0.237 | 0.797 | - | - | - | - |
| Previous Experience | -0.002 | 0.006 | -0.392 | 0.695 | -0.014 | 0.009 |
| Dominant >25m | -0.331 | 0.378 | -0.876 | 0.381 | -1.096 | 0.394 |
| Rank | -0.120 | 0.078 | -1.543 | 0.123 | 0.273 | 0.033 |

**Table S2.** Likelihood of focal vervet monkeys (*Chlorocebus pygerythrus*) obtaining maximum available food in an experimental trial at Lake Nabugabo, Uganda. Includes the focal’s rank as a predictor. Confidence intervals are set at 97.5%.

|  | ***B*** | **SE** | ***X^2^*** | ***P*** | **Lower CI** | **Upper CI** |
| --- | --- | --- | --- | --- | --- | --- |
| Intercept | 2.448 | 0.967 | - | - | - | - |
| **Dominant >25m** | **1.277** | **0.202** | **29.789** | **<0.001** | **0.811** | **1.649** |
| **Started at Nearest Platform** | **-0.480** | **0.193** | **6.092** | **0.014** | **-0.855** | **-0.099** |
| Rank | 0.122 | 0.089 | 2.302 | 0.129 | -0.044 | 0.289 |
| **Trial Number** | **-0.003** | **0.001** | **8.199** | **0.004** | **-0.006** | **-0.001** |

**
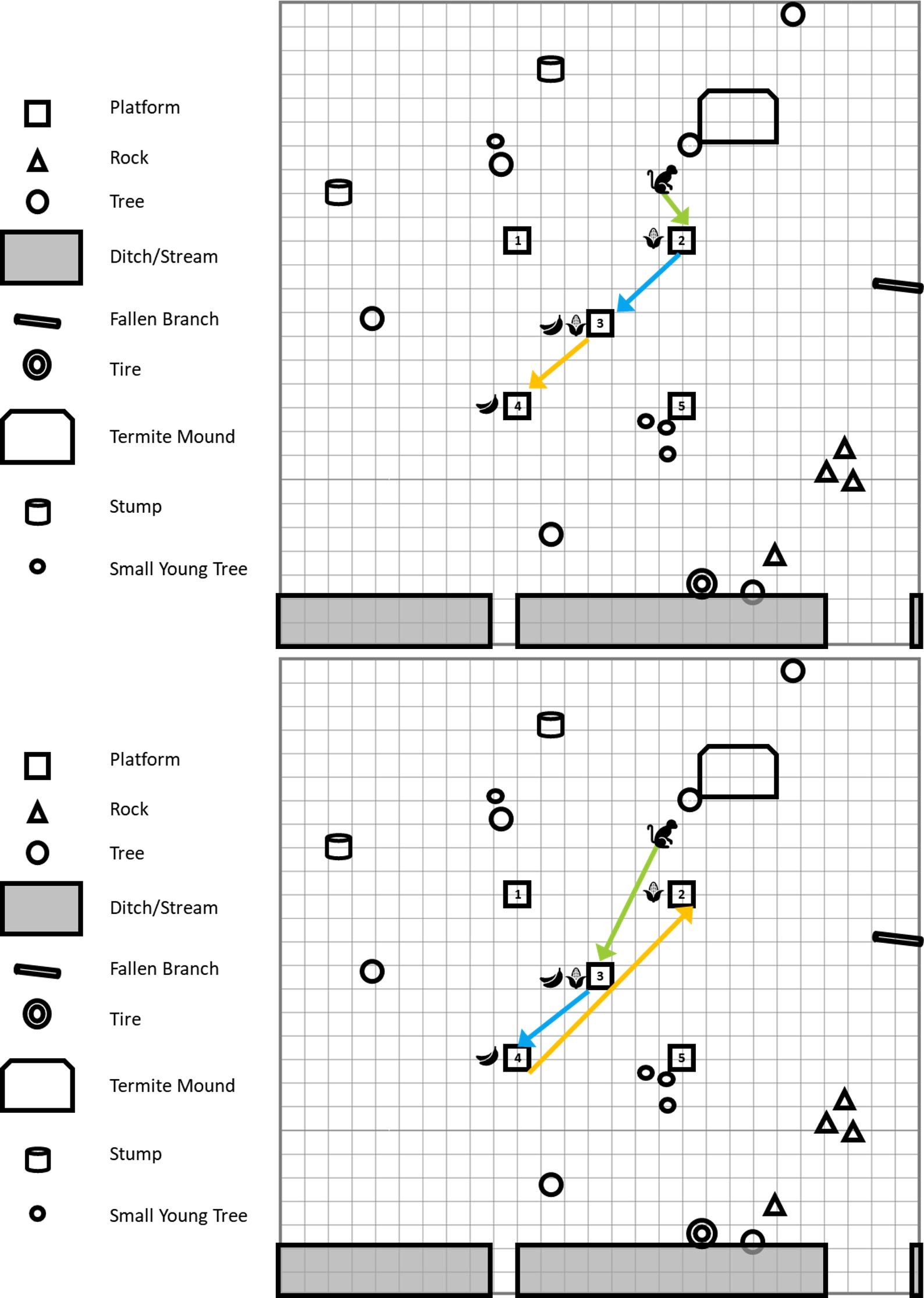
**

**Figure S1.** Figure of the experimental study site that was used in the field for recording the foraging decisions of vervet monkeys (*Chlorocebus pygerythrus*), as well as the location and movements of the vervet audience during trials near Lake Nabugabo, Uganda. Shapes represent various features of the study site while the numbered squares represent the platforms. The 27cm x 27cm included in Figure S1 represents an area of 27m x 27m surrounding the experiment site. In all trials, corn (the least-favoured reward) was placed at the platform closest to the individual anticipated to be the focal, in this case at Platform 2. Both corn and banana (the best reward) were always placed at the central Platform 3, and a piece of banana was placed at the platform furthest from the anticipated focal (Platform 4 in this example). The top figure shows an example of the experimental setup and possible route through baited platforms if the focal monkey chose to begin at their nearest platform (total distance traveled in the array - 10m). The bottom figure shows an example experimental setup and possible route through baited platforms if the focal monkey chose to begin at the central platform (total distance traveled in the array - 20 m). In these examples, platforms 1 and 5 would not be baited.
